# MSCs can be a double-edged sword in tumorigenesis

**DOI:** 10.3389/fonc.2022.1047907

**Published:** 2022-11-10

**Authors:** Lu Zhang, Junyu Xiang, Fang Zhang, Limei Liu, Chongling Hu

**Affiliations:** ^1^ Oncology Laboratory, Chongqing Key Laboratory of Translational Research for Metastasis and Individualized Treatment, Chongqing University Cancer Hospital, Chongqing, China; ^2^ Department of Biomedical Materials Science, Third Military Medical University, Chongqing, China; ^3^ Hematological Oncology Center, Chongqing University Cancer Hospital, Chongqing, China

**Keywords:** mesenchymal stem cells, cancer immunotherapy, extracellular vesicles, innate immune cells, adaptive immune cells

## Abstract

Mesenchymal stem cells (MSCs) have been used to treat various diseases including Alzheimer’s disease and cancer. In particular, the immunomodulatory function of MSCs plays a major role in cancer therapy using stem cells. However, MSCs exert promotive and inhibitory effects on cancer. The immunomodulatory effects of MSCs in the tumor microenvironment (TME) are ambiguous, which is the primary reason for the different outcomes of MSCs therapies for tumors. This review discusses the use of MSCs in cancer immunotherapy and their immunomodulatory mechanisms in cancers.

## 1 Introduction

**Figure 1:**
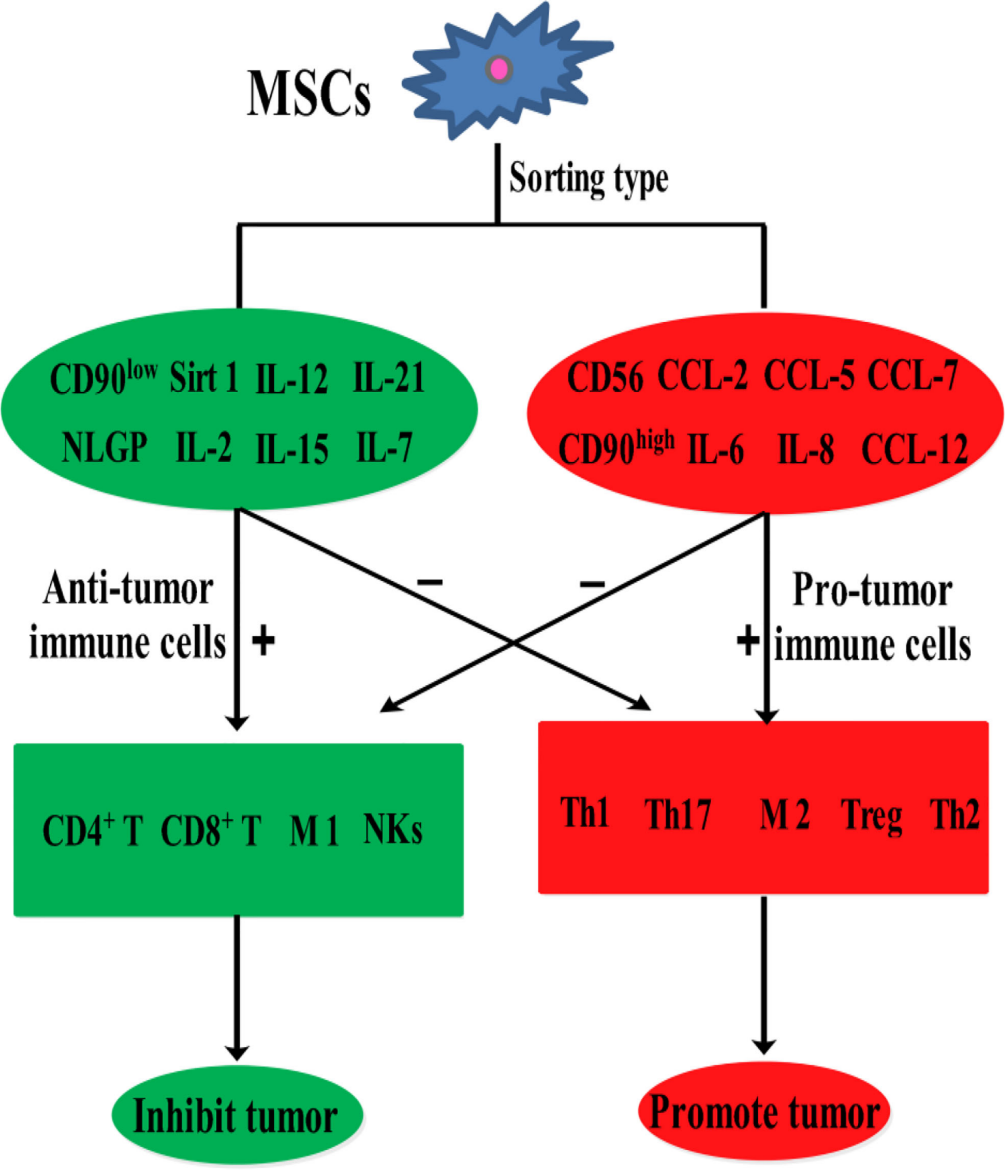
The effects of various MSCs subtypes on cancer.

**Figure 2:**
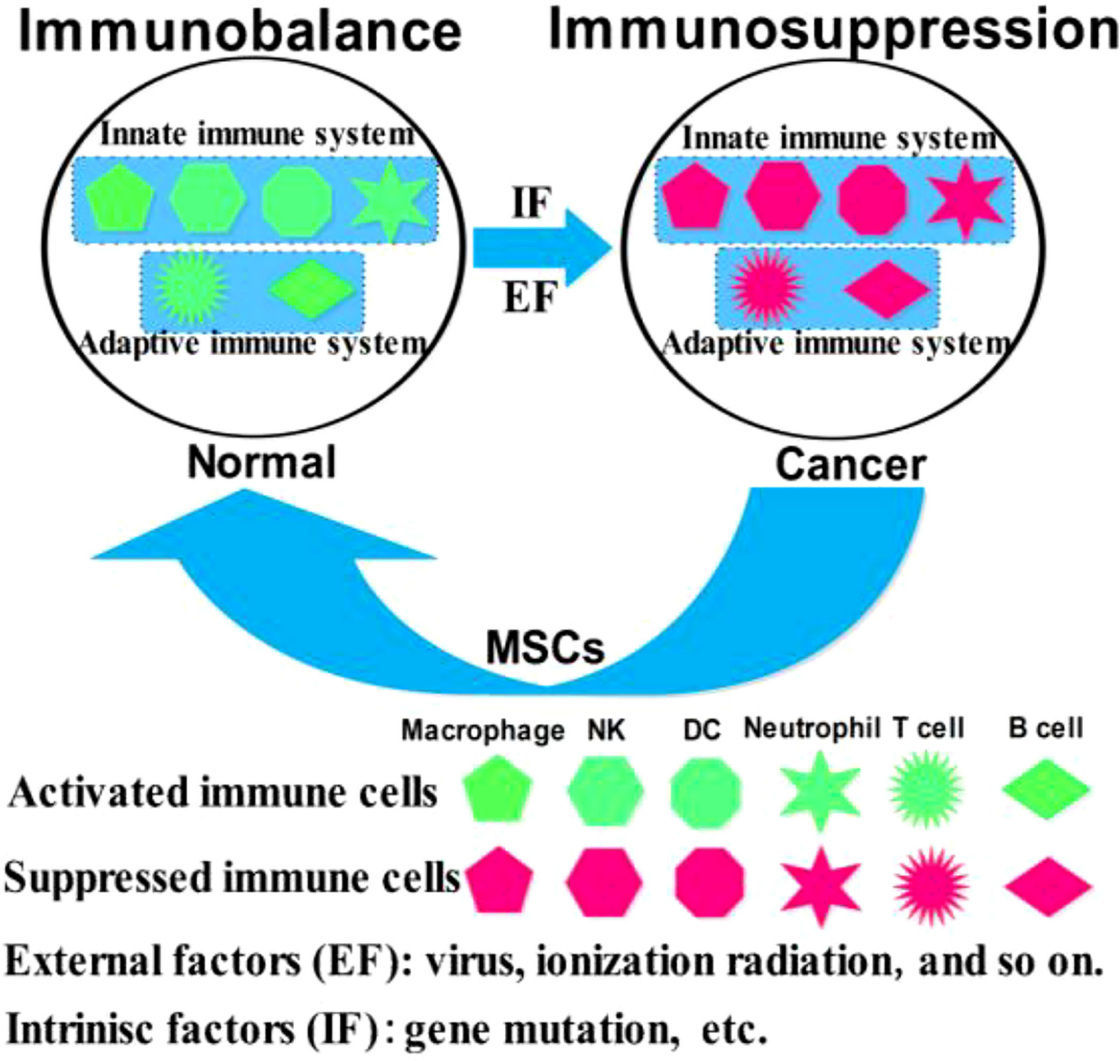
Schematic representation of the immunomodulatory roles of MSCs in cancer.

Mesenchymal stem cells (MSCs) are easily accessible stem cells with high differentiation potentials and immunomodulatory function. MSCs are mainly obtained from various tissues including bone marrow, umbilical cord, amniotic fluid, teeth, and fat tissues (1). MSCs are widely used to treat various disorders including neurodegenerative diseases (1), nerve injuries (2), and cancers (3). MSCs therapies have achieved good results for nervous system diseases. However, MSCs show different effects in tumor treatments, tumorigenesis, and development. For example, MSCs from umbilical cord stroma promote the proliferation and metastatic behaviors of breast cancer cell lines *in vitro*, such as retinoblastoma protein (Rb)^+^ MCF-7 and MDA-MB-231 (4). It has also been reported that microRNA-222/223 from MSCs promote Rb^+^ breast cancer recurrence and bone metastasis in a tumor-bearing mouse model (5). Additionally, a study using clinical samples found that cancer cells promotes breast cancer (Rb^+^) invasion and metastasis by the phagocytosis of MSCs (6). Recent studies have found that MSCs promotes the development of pancreatic cancer due to IL-6 by MSCs paracrine. Furthermore, the promoting effect of MSCs on tumor can be eliminated by knockout the expression of IL-6 in MSCs (7). Intriguingly, human umbilical cord stroma MSCs (hUC-MSCs) inhibited colon cancer *via* modulating the proportion of macrophages, which show that MSCs could regulate the polarization of macrophages (8). These studies indicate that MSCs therapy may promote cancer development and metastasis. This is largely because of the heterogeneity of MSCs and the heterogeneity of tumors. Indeed, different subsets of MSCs show varying immunomodulatory functions in tumors (**Figure 1**). For example, transplantation of CD90^low^ MSCs derived from mouse compact bone into a mouse model of ovarian cancer promotes the expression of interleukin-12 (IL-12), interleukin-21 (IL-21), interferon-γ (IFN-γ), and the pro-inflammatory factor chemokine (C-X-C motif) ligand 10 (CXCL10), and inhibited the expression of anti-inflammatory factors, including interleukin-10 (IL-10) and C-C chemokine ligand-5 (CCL-5), thereby suppressing tumor growth and improving survival (9). Reprogrammed interleukin-7 (IL-7)-IL-12-MSCs are reported to promote the activation of CAR-T immune cells and the release of IFN-γ and tumor necrosis factor-α (TNF-α), which markedly enhanced colorectal cancer cell death (10). However, interleukin-17 (IL-17) significantly promotes the immunosuppressive function of MSCs by inducing the expression of IFN-γ and TNF-α (11). IL-17 has been reported to mediate immunosuppression by enhancing the expression of programmed death 1 (PD-1) in MSCs *via* inducible nitric oxide synthase (iNOS) (12). Therefore, MSCs could suppress tumor development by activating immune cells or promote tumor development by suppressing immune cells (**Figure 2**).

## 2 The inhibitory effects of MSCs in cancers

Although MSCs are used in cancer therapy, some studies show that they have the potentials to inhibit or promote tumor development. For instance, human adipose-derived MSCs (Hu-ADSCs) promote epithelial-mesenchymal transition (EMT) in MCF-7 cells *via* the TGF-β/SMAD and PI3K/AKT pathways in the TME (13). Studies have shown that the secretion of interleukin-8 (IL-8) and interleukin-6 (IL-6) by human umbilical cord-derived MSCs (hUCMSCs) activates the expression of IL-8 and IL-6 in MCF-7 cells through autocrine signaling and induction of CD44^+^/CD24^-^ cells, thereby promoting MCF-7 cell migration *in vitro* and *in vivo* (14). Hypoxic preconditioning enhances the expression of miR-21-5p-derived from MSCs extracellular vesicles (MSC-EVs), which can promote lung cancer development by suppressing apoptosis and promoting M2 macrophage polarization (15). Human menstruation blood-derived MSCs (hMBSCs) are reported to mediate their anti-cervical cancer effects, *in vitro* and *in vivo*, through the TGF-β1/JNK/p21 signaling pathway (16). It is reported that intravenous injection of human amniotic MSCs (hAMSCs) into mice bearing HepG2, a hepatocellular carcinoma cell line, induces HepG2 apoptosis and significantly suppresses its proliferation. Further antibody array analysis showed that the hAMSCs overexpressed dickkopf-3 (DKK-3), dickkopf-1 (DKK-1), and insulin-like growth factor binding protein-3 (IGFBP-3). More importantly, hAMSCs and their conditioned media exhibit similar anti-tumor effects *in vitro*, suggesting that the anti-tumor effects of hAMSCs may be mediated by hAMSCs-derived cytokines including DKK-3, DKK-1, and IGFBP-3 (17). It is reported that lipoxin A4, as an endogenous lipoxygenase-derived eicosanoid mediator, could reverse the mesenchymal phenotype of pancreatic cancer and suppress its invasion and metastasis by inhibiting autocrine TGF-β1 signaling, and that therapeutically targeting this process may prevent pancreatic cancer metastasis (18). These studies highlight the different potential anti-cancer effects of MSCs, which may be because MSCs have a variety of immunomodulatory functions in the TME (**Table 1**).

**Table 1:** The cancer immunomodulatory effects of various MSCs.

Cells	Immune state	Molecular mechanism	Results	Ref
PC-MSCs	immunosuppression	IFN-γ/TNF-α-PD-L1/PDL2	promotion	(19)
	immunosuppression	IL-8/PD-L1	promotion	(20)
GC-MSCs	immunosuppression	CD4^+^ -T-PD-L1	promotion	(21)
MSCs	immunoactivation	Macrophages are re-coded as regulatory cells involved in phagocytosis, thus inhibiting the production of pro-inflammatory cytokines.	inhibition	(22)
CMSCs	immunosuppression	Ly6G^+^-MDSCs inhibits the proliferation of T cells.	promotion	(23)
	immunosuppression	PD-1/PD-L1 pathway.	promotion	(24)
BMSCs	immunoactivation	myeloid-derived suppressor cells.	inhibition	(25, 26)
	immunoactivation	Hsa-miR-23b-3p maintains the balance of Th17/Treg.	inhibition	(27)

PC-MSCs, prostate cancer-infiltrating MSCs; GC-MSCs, gastric cancer mesenchymal stem cells; MSCs, mesenchymal stem cells; CMSCs, cancer-educated mesenchymal stem cells; BMSCs, bone marrow-derived mesenchymal stem cells.

In cancer inhibition (**Table 2**), MSCs are reported to inhibit the production of pro-inflammatory factors, cause the conversion of macrophages into phagocytic regulatory cells, and activate the immune microenvironment of colorectal cancer (22). Bone marrow MSCs (BMSCs) are reported to suppress tumorigenesis by inhibiting the production and proliferation of myeloid-derived suppressor cells (MDSCs) (25). Interestingly, when BMSCs were transplanted into a mouse model of hepatoma to treat ascites, tumor growth was inhibited and the survival time of the mice was prolonged, probably because BMSCs inhibited the production and proliferation of MDSCs (26). Hsa-miR-23b-3p from BMSCs is reported to inhibit PI3k/Akt/NF-κB signaling, to maintain the T helper type 17 cells (Th17)/regulatory T cells (Treg) balance, and to activate the tumor immune microenvironment, thereby suppressing the development of intracranial tumors (27).

**Table 2:** The inhibitory effect of MSCs in cancers.

Cells	Tumors	Mode of action	Results	Ref
BMSCs	Intracranial aneurysm	EVs	Inhibition	(27)
	Cervical cancer	EVs	Inhibition	(28)
	Colorectal cancer	EVs	Inhibition	(29)
	Ovarian cancer	Paracrine	Inhibition	(9)
hMBSCs	Cervical cancer	Paracrine	Inhibition	(16)
Hu-ADSCs	Neuroblastoma	Paracrine	Inhibition	(30)
MSCs	Colorectal cancer	EVs	Inhibition	(22)
	Glioma	EVs	Inhibition	(31)
hUCESCs	Breast cancer (Rb^+^)	Cell to cell	Inhibition	(32)
hAMSCs	Hepatocellular carcinoma	Paracrine	Inhibition	(17)

hUCESCs, human uterine cervix-derived MSCs; hAMSCs, human amniotic mesenchymal stem cells; hMBSCs, human menstrual blood-derived stem cells; Hu-ADSCs, human adipose-derived stem cells.

## 3 The promoting effects of MSCs in cancers

In cancer promotion, prostate cancer-infiltrating MSCs are reported to mediate their immunosuppressive effects by suppressing the proliferation of T cells in a dose-dependent manner, and to upregulate the cell surface levels of programmed death ligand-1 (PD-L1)/programmed death ligand-2 (PD-L2) *via* IFN-γ/TNF-α signaling (19). Secretion of IL-8 by gastric cancer associated MSCs (MSCs originated from human GC-MSCs) promotes the expression of PD-L1 by gastric cancer cells, causing resistance to cytotoxic CD8^+^ T cells. Studies have shown that PD-L1 expression in gastric cancer regulates the production of c-Myc to promote tumor development through STAT3/mTOR signaling (20). GC-MSCs promote the activation of CD4^+^ T cells, which in turn, promote PD-L1 expression in GC-MSCs *via p*-STAT3 signaling, thereby promoting tumor growth (21). BMSCs home to the TME in response to chemokines and cytokines secreted by cancer cells, which are ‘educated’ by the TME and promote the generation of Ly6G^+^ MDGCs that inhibit T cells proliferation to suppress the tumor immune microenvironment (23). In a mouse model of multiple myeloma, BMSCs are reported to inhibit T cells immune response by means of the PD-1/PD-L1 signaling pathway, thereby promoting the development of multiple myeloma, and markedly shortening mouse survival (24). Thus, MSCs can promote or suppress immune function through various immunomodulatory mechanisms, thereby influencing tumor development (**Table 3**).

**Table 3:** The promoting effect of MSCs in cancers.

Cells	Tumors	Mode of action	Results	Ref
ADSCs	Lung cancer	Paracrine	Promotion	(33)
BMSCs	Multiple myeloma	Paracrine	Promotion	(24)
	Lung cancer	EVs	Promotion	(15)
Hu-ADSCs	Breast cancer (Rb^+^)	Paracrine	Promotion	(13)
hUCMSCs	Lung adenocarcinoma	EVs	Promotion	(34)
UC-MSCs	Breast cancer	Paracrine	Promotion	(14)
	Breast cancer (Rb^+^)	EVs	Promotion	(5, 35)
MSCs	Lung cancer	Paracrine	Promotion	(36)
	Breast cancer (Rb^+^)	Cell to cell	Promotion	(6)

ADSCs, adipose-derived mesenchymal stem cells; Hu-ADSCs, human adipose-derived stem cells; hUCMSCs, UC-MSCs, human umbilical cord stroma-derived MSCs.

## 4 The immunoregulatory role of MSCs and immune cells in the TME

**Figure 3:**
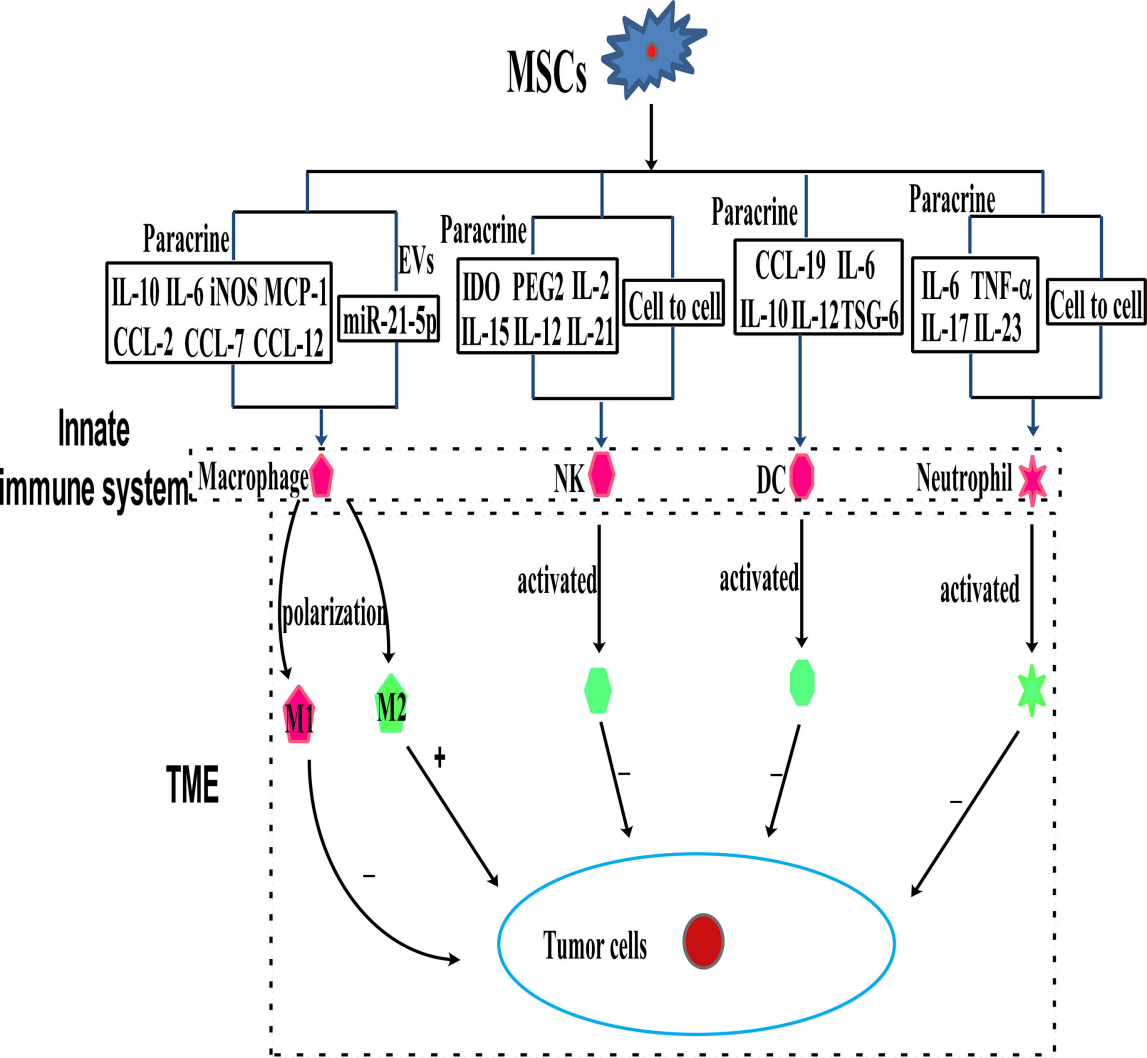
MSCs can modulate innate immune cells through the mechanism of paracrine cytokines, EVs, and cell to cell interaction.

**Figure 4:**
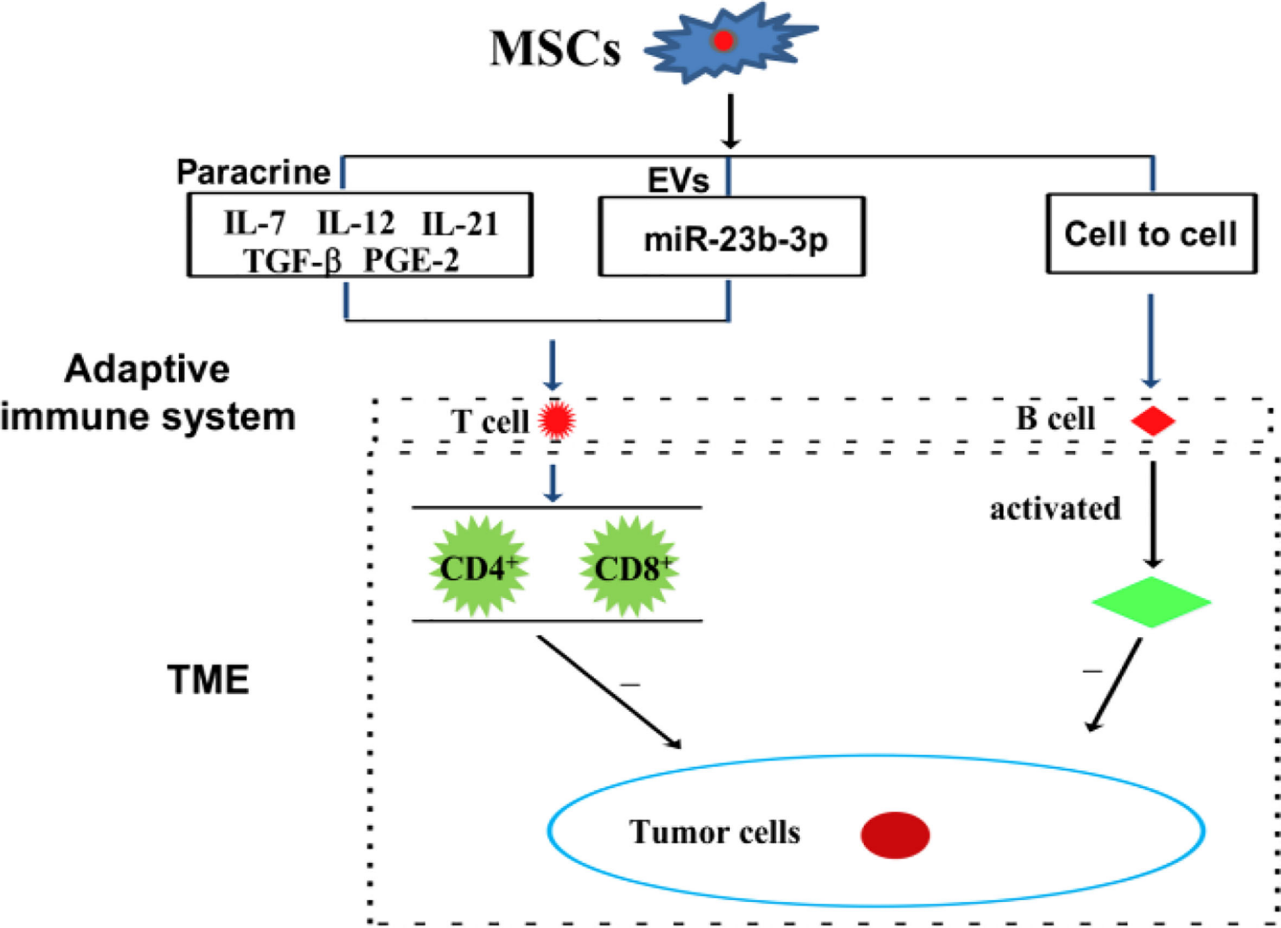
MSCs can modulate adaptive immune cells through the mechanism of paracrine pathway, EVs, and cell to cell interaction.

Immune cells, including innate and adaptive immune cells, play an important role in the TME. In particular, TME is mainly composed of tumor cells, immune and inflammatory cells around them, tumor-related fibroblasts, and nearby interstitial tissues, microvessels, and various cytokines and chemokines. It is a complex and comprehensive system, which can be divided into immune microenvironment dominated by immune cells and non-immune microenvironment dominated by fibroblasts. Among them, Innate immune cells include macrophages, natural killer cells (NKs), neutrophils, and dendritic cells (DCs). Adaptive immune cells include lymphoid T cells and B cells. However, MSCs have a variety of immunomodulatory effects on innate and adaptive immune cells (**Figures 3**, **4**), through several pathways.

### 4.1 The effect of MSCs on innate immune cells in the TME

#### 4.1.1 MSCs and macrophages

Macrophages as effector cells of the innate immune system play a vital role in mediating host anti-cancer responses by initiating and participating in immune responses (37, 38). The primary functions of macrophages in mediating tumor regression are phagocytosis, direct lysis of cancer cells, and secretion of cytokines with direct or indirect tumoricidal activities. Macrophages are divided into two subtypes containing M1 and M2. M1 macrophages activate anti-tumor effects and protect from carcinogenesis. There is evidence that the TME contains M2 macrophages, which inhibit anti-tumor immune responses (39–41). MSCs can exert their anti-tumor effects by interacting with macrophages in the TME (39, 41), and they have been reported to bias macrophages toward the “anti-inflammatory” M2 subtype, which is characterized by elevated IL-10 levels and reduced expression of iNOS and IL-12 (39, 41–43). However, it is reported that intraperitoneal injection of MSCs into a mouse model of colorectal cancer did not fully repair inflammation-driven intestinal mucosal lesions, but the MSCs accumulated in the abdominal cavity, accompanied by T cells and macrophages. The macrophages treated with MSCs exhibit the M2 phenotype, with the levels of IL-10 and iNOS elevated, while those of IFN-γ, IL-6, and TNF-α, were reduced (44). It is reported that the iNOS, MCP-1,and IL-6 secreted by M1 condition medium MSCs could polarize the infiltrating tumor-associated macrophages (TAM) into M2 macrophages, thereby causing immunosuppression and promoting tumor development (45). The EVs secreted by MSCs in ischemic preconditioning may promote the M2 polarization of macrophages and the growth and invasion of non-small lung cancer cells (NSCLC) through miR-21-5p (15). Similarly, human placental MSCs play an immunosuppressive role by transforming pro-inflammatory M1 macrophages into anti-inflammatory M2 macrophages (46). Additionally, MSCs produce CCL-5, which binds to CCR-5, causing the secretion of colony-stimulating factor-1 (CSF-1) again, and CSF-1 binding to CSF-1 receptors in MSCs to promote the recruitment of macrophages (47). Tumor-educated MSCs release large amounts of chemokines, including C-C chemokine ligand-2, C-C chemokine ligand-7, and C-C chemokine ligand-12, thereby accelerating the recruitment of CCR-2 dependent monocytes and macrophages to the tumor, which finally promotes tumor growth (48).

Thus, MSCs-mediated transition of macrophages from M1 to M2 subtype may be closely associated with immunoregulation in the TME.

#### 4.1.2 MSCs and NKs

NKs are the main effector cells of the innate immune system, which directly kill virus-infected and stressed cells as well as tumor cells in an MHC-independent manner (49). NKs are a part of the host natural defense mechanism. They use C-type lectin molecules for target recognition. Therefore, NKs are thought to be involved in tumor surveillance. It is reported that the overexpression of Sirtuin-1 by MSCs recruits NKs to the TME and effectively inhibits the growth of breast cancer (Rb^+^) cells (50). Interestingly, MSCs from various tissues have different immunosuppressive effects on NKs. Studies have shown that tumor-derived MSCs are more immunosuppressive than normal MSCs, which may be caused by the differential expression of NCAM (CD56) (51). It is reported that the immunomodulatory effects of MSCs vary across NK cell lines. For instance, MSCs suppress IFN-γ secretion by the NK cell line, KHYG-1, but not by NK-92 (another NK cell line). Whereas MSCs are more sensitive to NK-92 than KHYG-1. Interestingly, the immunosuppression of MSCs is regulated by indoleamine 2,3-dioxygenase (IDO) and prostaglandin E2 (PGE-2), which are secreted by MSCs (52). Transcriptional activator with a PDZ motif (TAZ)-expressing MSCs inhibit NKs receptor stimulation, signal ligand and NKs cytotoxicity *via* the mechanism of cell-to-cell contact regulation, and have a strong immunosuppressive effect on NKs (53). On the contrary, MSCs derived from Wharton’s Jelly derived activates NKs by secreting cytokines, including IL-2, IL-12, interleukin-15 (IL-15), and IL-21, with the latter influencing immune responses by driving the secretion of IFN-γ and TNF-α (54).

Although the mechanisms underlying the interaction between MSCs and NKs in tumors are not clear, above studies indicate that the paracrine function of MSCs in the TME may be involved.

#### 4.1.3 MSCs and DCs

DCs are the main type of antigen-presenting cells (APCs) and an important component of innate immunity. In cancer, danger signals promote DCs activation and/or maturation culminating in an antigen-specific T cells response that is necessary for pathogen clearance and killing cancer cells.

MSCs mainly affect the immune regulation of lymphocytes by regulating the transmission of DCs antigen. Several studies have focused on the regulation of DCs differentiation by MSCs. For example, during the maturation of DCs, the supernatant from MSCs inhibits CD83 expression, suppresses IL-12 production, and interferes with endocytosis (55). Additionally, it is reported that MSCs block the differentiation of CD14^+^/CD1a progenitor cells into dermal/interstitial DCs, but they do not affect the production of CD1^a+^ Langerhans cells. It is reported that MSCs completely inhibit the differentiation of monocytes into immature DCs by secreting IL-6, macrophage colony-stimulating factor (M-CSF), or other soluble factors (56). BMSCs have been reported to partially inhibit the differentiation of DCs into bone marrow progenitor cells by secreting IL-6 (57). IL-10, an immunosuppressive cytokine, influences the differentiation and maturation of DCs *via* JAK/STAT signaling, and MSCs are reported to inhibit DCs maturation by stimulating IL-10 secretion and JAK1/STAT3 signaling. In addition to IL-10, the secretion of TNF-α stimulating gene-6 (TSG-6) by MSCs has been shown to inhibit the activation of MAPK and NF-κB signaling during LPS-induced DCs maturation (58). These results suggest that MSCs maintain an immature or semi-mature DCs phenotype. Interestingly, studies have shown that BMSCs also block DCs migration in response to chemokine (C-C motif) ligand-19, thereby interfering with antigen presentation by DCs (59, 60). However, MSCs are less directly involved in tumor immune regulation, and more indirectly participate in tumor immune regulation *via* regulating DCs. For example, tumor-associated MSCs are reported to modulate the expression of cysteinase *via* the IL-10/STAT3 signaling pathway, thereby inhibiting the production of cysteine by DCs and suppressing the proliferation of naïve T cells (61).

These findings indicate that MSCs interfere with the three main functions of DCs, namely, the upregulation of antigen presentation and co-stimulatory molecules, their antigen presentation capacity, and migration ability of specific antigens. However, further studies are needed to determine how MSCs modulate DCs in tumors.

#### 4.1.4 MSCs and neutrophils

Neutrophils are short-lived effector cells of the innate immune system and they play major roles in the activation, localization, and expression of adaptive immune responses (62). There are two important aspects of the role of neutrophils in cancer: (1) the main purpose of neutrophilic functions is killing infectious microorganisms, and neutrophils were not evolutionarily programmed to fight or support cancer; (2) neutrophils are a plastic and diverse population of cells, which either support or interfere with cancer development and metastasis. Reprogramming of neutrophils by tumors results in phenotypic modulation that reshapes these functions to support tumor progression.

GC-MSCs inhibit the chemotaxis, survival, activation, and function of neutrophils through the IL-6/STAT3/ERK1/2 signaling pathway and promote the development of gastric cancer (63). MSCs promote the storage of neutral fat in neutrophils, which enters breast metastatic cells through the macrophage-lysosome pathway, thereby providing tumor cells with energy for survival and proliferation (64). MSCs induce neutrophil activation *via* AKT/p38 signaling, secrete the inflammatory factors IL-17, IL-23, and TNF-α, and promote the growth and metastasis of gastric cancer (65). When co-cultured with MSCs, neutrophils are protected by IL-6 secretion by MSCs, and participate in STAT3 signaling. Surprisingly, TNF-α-activated MSCs secrete the high levels of CXCR2 ligands, such as CXCL1, CXCL2, and CXCL8, which mediate the recruitment of neutrophils by MSCs. This is mainly because the chemokine receptor CCR-2 that is highly expressed in neutrophils, blinds to CXCR2. In turn, the neutrophils promote cancer metastasis (66). These studies provide the guidance for the immunomodulatory mechanism of TME mediated by neutrophils regulated by MSCs *via* paracrine pathway.

### 4.2 The effects of MSCs on adaptive immune cells in the TME

#### 4.2.1 MSCs and lymphocyte T cells

T cells are the main cellular effectors of adaptive immunity, which play major roles in antigen-specific and memory-related homologous immunity (67). T cells have two of the major functions in mounting a response against cancer cells, which are the recognition and direct killing of cancer cells, a process known as cell-mediated cytolysis, and the production and secretion of cytokines that induce activation and proliferation of other effector cell populations. There are two primary T cells subgroups, namely CD4^+^ and CD8^+^ T cells, which are involved in developing an effective immune response against cancers.

In a mouse model of multiple myeloma, BMSCs have been reported to suppress T cell-mediated immune responses *via* the PD-1/PD-L1 signaling pathway, including by inhibiting the proliferation of CD4^+^ T cells, reducing the Th1/Th17 ratio, and increasing the levels of Th2 and Treg cells. Meanwhile, the cytokines of the corresponding T cells subsets also changed (24). In a Balb/c nu/nu tumor transplantation model, CD4^+^ T cells are reported to upregulate PD-L1 expression in gastric cancer-derived MSCs (GC-MSCs) *via* the *p*-STAT3 signaling pathway, and to activate the PD-1/mTOR signaling pathway, thereby promoting gastric cancer growth (21). Interestingly, exosomal miRNA derived from BMSCs, hsa-miR-23b-3p, targets Krüppel-like factor 5 (KL5) by inhibiting PI3k/Akt/NF-κB signaling and maintaining the balance of Th17/Treg, thereby inhibiting the development of intracranial aneurysms (21). Recombinant IL-7/IL-12 MSCs are reported to enhance the ability of CAR-T cells to attack colorectal cancer (10). In cervical cancer, MSCs protect cancer cells from attack by cytotoxic T cells by downregulating the expression of HLA class I (68). Tumor-derived MSCs inhibit T cells proliferation and block cysteine transport to T cells through DCs cells (61). Amazingly, nontoxic neem leaf glycoprotein (NLGP) has been shown to upregulate cysteine expression by suppressing IL-10 secretion by TC-MSCs, thereby restoring the proliferation and effector function of T cells in the TME (69). MSCs secrete inflammatory cytokines, including CCL-5 and IL-17B, and which promote tumor invasion and metastasis (70). IL-21 secreted by MSCs could effectively inhibit malignant B lymphoma by inducing T cells and NKs, and by suppressing immunosuppressive cells (71).

A recent study found that through paracrine function, MSCs play different regulatory roles on different T cells subtypes. MSC-secreted PGE-2 and TGF-β could induce CD4^+^/CD25^+^/FoxP3^+^ T cells (72, 73). Similarly, MSC-secreted HLA-G5 helps to inhibit the proliferation of allogeneic T cells and CD4^+^/CD25/HighFoxP3^+^ Tregs (74). Findings from a mouse model of encephalomyelitis indicate that MSCs release CCL-2 by inhibiting the STAT3 signaling pathway, thereby inhibiting the activation of CD4^+^/Th17 cells (75). Additionally, MSCs inhibit Th17 cells differentiation at least in part, by secreting PGE2 and IDO (76). In addition to their direct effect on T cells, MSCs also influence T cells by regulating innate immune cells, including macrophages and DCs, through MSCs-derived paracrine factors. The interaction between co-stimulatory ligands and TCR on the surface of T cells is necessary for T cells activation. Therefore, the soluble factors produced by MSCs can affect the expression of co-stimulatory ligands in APCs, thereby regulating T cells. For example, MSCs affect their immunomodulatory functions by influencing macrophage polarization, which control lymphocyte T cells differentiation (77). Thus, paracrine factors from MSCs immunomodulate the TME by directly and/or indirectly, transmitting regulatory signal to lymphocyte T cells.

#### 4.2.2 MSCs and lymphocyte B cells

B cells are another major adaptive immune cell types involved in antigen presentation and antibody production (78, 79). B cells mount an anti-cancer response by generating tumor-specific antibodies. Tumor-bound antibodies help to locate a tumor by serving as recognition molecules to which effector cells bind through their constant fragment (Fc) receptor. A specialized feature of certain cytotoxic effector cells is their expression of receptors for the Fc region of IgG molecules. When tumor cells are coated with IgG, effector cells with Fc receptors bind to the target cell and kill it. ADSCs promote the survival of resting B cells in a contact-dependent manner. However, induced regulatory B cells are independent of helper T cells (80). In a mouse model of head and neck cancer, hematopoietic stem cells combined with MSCs promoted lymphoid B and T cells tumor infiltration, and effectively inhibited tumor development (81). ADMSCs inhibit B cells proliferation depending on the presence of T cells (80).

However, it is not clear how the paracrine activity of BMSCs affects lymphocyte B cells in the TME, which warrants further investigation.

## 5 Immunoregulatory roles of EVs-derived MSCs in the TME

MSCs are thought to mainly mediate their immunomodulatory functions through paracrine signals. Several recent studies have shown that secretory EVs contribute to this regulatory effect. Additionally, MSCs-secreted EVs are considered to be key paracrine factors. MSC-EVs, which include microcapsules and small EVs are a heterogeneous group of lipid membrane-encapsulated nanoparticles containing various biomolecules, RNAs (like mRNAs and miRNAs), and proteins (such as membrane receptors, enzymes, cytokines, and growth factors) (82). MSC-EVs influence cell-cell interactions by transferring bioactive molecules (locally or remotely) from signaling cells to signal-receiving cells (83, 84). EVs obtain their contents from parental MSCs, so EVs-derived from MSCs have similar immunomodulatory properties (85, 86). Thus, MSCs could mediate their immunomodulatory effects through the release of EVs.

It is reported that BMSCs-derived EVs suppress immune function in multiple myeloma by activating MDSCs through the STAT3/STAT1 signaling pathway, thereby promoting the development of multiple myeloma (87). ADMSCs-derived EVs carrying miR-10a promote the differentiation and immune response of Th17 cells and Tregs, but suppress Th1 differentiation, thereby indirectly enhancing the immunomodulatory function of MSCs (88). The membrane sac produced by human MSCs with cytochalasin B-induced IL-2 overexpression could stimulate cytotoxic CD8^+^ T cells in triple negative breast cancer (Rb^+^) cells (89). In breast cancer (Rb^+^), MSCs-derived EVs promote the differentiation of myeloid cells into M2 macrophages, resulting in immunosuppression and enhanced tumor growth (90). MSCs-derived EVs carrying microRNA-15a inhibit immune escape by colorectal cancer cells by regulating the KDM4B/HOXC4/PD-L1 axis, thereby inhibiting tumor development (91). Surprisingly, the EVs secreted by Wharton’s Jelly-derived MSCs promote T cells inhibition through PD-L1 (92). These reports show that MSC-EVs can be used as an potential tool in cancer immunotherapy (93).

Compared with MSCs, acellular EVs have low-immunogenicity, the ability to cross biological barriers, and strong potential for tumor. But overcoming the problem of quality heterogeneity still is big problem (86, 94). It is relatively easy to modify EVs in order to improve their effective content and surface availability, thereby improving their therapeutic potential. Based on these advantages, MSC-EVs are expected to develop into effective alternatives to stem cell immunotherapy.

## 6 Perspectives

Because of their high differentiation potentials and immunomodulatory function, MSCs have promising application potentials in regenerative medicine and cancer therapy. However, in cancer therapy, MSCs exhibit varying therapeutic effects. Indeed, clinical trials on the use of MSCs in cancer therapy (**Table 4**) have yielded unexpected results. This may be due to the effects of the tumor immune microenvironment, in which immune cells are inhibited by various factors, thereby creating an environment that is conducive for tumor growth. MSCs influence tumor immune regulation by enhancing or suppressing immune activation, thereby influencing the therapeutic effects on various tumors differently. Because of their heterogeneity, the effects of various MSCs subtypes in the tumor immune microenvironment, including their effects on anti- or pro-tumor immune cells. (**Figure 1**). Thus, preclinical MSCs typing may be necessary to identify the MSCs with anti-cancer potentials. Additionally, tumor heterogeneity, coupled with the complex immune microenvironment, significantly complicates cancer treatment. Therefore, using MSCs as mediators of immunomodulatory activity against tumors offers a novel strategy for cancer immunotherapy.

**Table 4:** Anti-cancer MSCs in registered clinical trials (Clinicaltrials. gov, National Institutes of Health) as of August 2021.

Cells	Tumors	Phase	Status	NCT	Location
Ah-BMSCs	Prostate Cancer	Phase 1 (N=7)	Terminated	NCT01983709	Maryland, US
MSCs	Ovarian cancer	Phase 1 (N=5)	Completed	NCT02530047	Texas, US
HB-adMSCs	Pancreatic cancer	Not available	Not available	NCT04087889	Texas, US
	Hematological Malignancies	Phase 1 (N=6)	Completed	NCT03106662	Ankara, Turkey
	Hematological Malignancies	Phase 1 (N=30)	Completed	NCT00504803	Liege, Belgium
	Head and neck cancer	Not applicable (N=20)	Recruiting	NCT02331134	Colorado, US
MSCs	Ovarian	Phase 1 Phase 2 (N=57)	Recruiting	NCT02068794	Minnesota, US
	Head and neck cancer	Phase 2 (N=120)	Recruiting	NCT04776538	Copenhagen, Denmark
	Head and neck cancer	Phase 1 (N=12)	Unknown	NCT02079324	Seoul, Korea
	Leukemia	Phase 1 (N=98)	Completed	NCT00498316	Texas, US

Ah-BMSCs, allogeneic human bone marrow-derived mesenchymal stem cells; HB-adMSCs, allogeneic adipose-derived mesenchymal stem cells.

## 7 Conclusions

MSCs have promising potential applications in stem cell therapy for cancers. However, because MSCs can mediate immunoactivation or immunosuppression in the TME, it is necessary to determine their immunomodulatory functions in various tumor types in order to ensure their effectiveness against cancers. What is more important is to determine the relationship between MSCs and different immune cells and how to affect the activity of immune cells, so as to inhibit or promote tumor development. Therefore, it is of great significance to understand the immunomodulatory mechanism of MSCs in cancer therapy.

## Author contributions

LZ conceived and wrote the paper. JX, FZ, and CH collected some literatures about MSCs in tumor. LL offer some suggestion and thoughts. All authors contributed to the article and approved the submitted version.

## Funding

This work was supported by grants cstc2020jcyj-msxmX0064 from the Natural Science Foundation of Chongqing.

## Conflict of interest

The authors declare that the research was conducted in the absence of any commercial or financial relationships that could be construed as a potential conflict of interest.

## Publisher’s note

All claims expressed in this article are solely those of the authors and do not necessarily represent those of their affiliated organizations, or those of the publisher, the editors and the reviewers. Any product that may be evaluated in this article, or claim that may be made by its manufacturer, is not guaranteed or endorsed by the publisher.
